# Neuronal Responses to Physiological Stress

**DOI:** 10.3389/fgene.2012.00222

**Published:** 2012-10-26

**Authors:** Konstantinos Kagias, Camilla Nehammer, Roger Pocock

**Affiliations:** ^1^Biotech Research and Innovation Centre, University of CopenhagenCopenhagen, Denmark

**Keywords:** stress responses, neuronal homeostasis, aging, developmental stress

## Abstract

Physiological stress can be defined as any external or internal condition that challenges the homeostasis of a cell or an organism. It can be divided into three different aspects: environmental stress, intrinsic developmental stress, and aging. Throughout life all living organisms are challenged by changes in the environment. Fluctuations in oxygen levels, temperature, and redox state for example, trigger molecular events that enable an organism to adapt, survive, and reproduce. In addition to external stressors, organisms experience stress associated with morphogenesis and changes in inner chemistry during normal development. For example, conditions such as intrinsic hypoxia and oxidative stress, due to an increase in tissue mass, have to be confronted by developing embryos in order to complete their development. Finally, organisms face the challenge of stochastic accumulation of molecular damage during aging that results in decline and eventual death. Studies have shown that the nervous system plays a pivotal role in responding to stress. Neurons not only receive and process information from the environment but also actively respond to various stresses to promote survival. These responses include changes in the expression of molecules such as transcription factors and microRNAs that regulate stress resistance and adaptation. Moreover, both intrinsic and extrinsic stresses have a tremendous impact on neuronal development and maintenance with implications in many diseases. Here, we review the responses of neurons to various physiological stressors at the molecular and cellular level.

## Introduction

**Figure 1:**
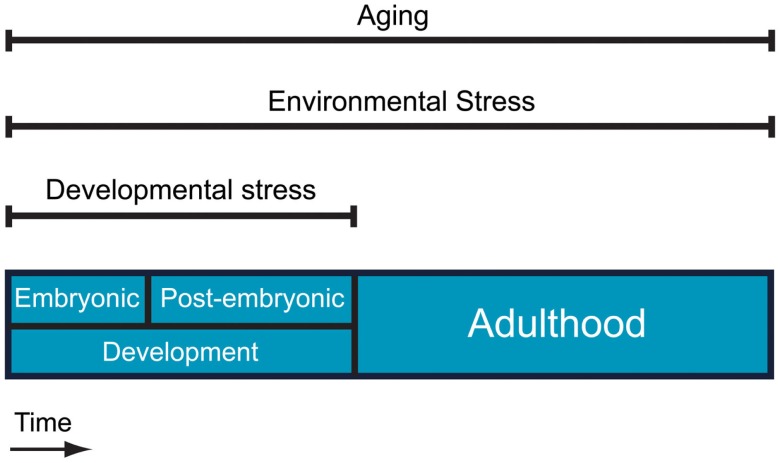
**Different aspects of physiological stress**. Aging and environmental stress are present throughout life whereas intrinsic developmental stress applies only during embryonic and post-embryonic development.

Stress is an inherent component of the natural world that applies to virtually all the biological systems. Biological stress signifies any condition that forces living systems away from a physiological steady state, and its impact is closely connected to the nature of elements that shape living organisms. As stress can be applied to many different levels of biological organization, the term has been used in many different contexts to date. “Physiological stress” is referred to as the primary biological stress and can be defined as any external or internal condition that challenges the homeostasis of a cell or an organism. Taking into account the different possible sources of biological stress, we can conceive three different aspects of physiological stress: environmental stress, intrinsic developmental stress, and aging (Figure 1).

### Environmental stress

Biological systems are designed to develop and live in a variety of changing environments. During evolution many different adaptations have been developed to provide organisms with the ability not only to survive but also to reproduce under different, often hostile conditions. Such adaptations are associated with specific structures and behaviors tailored to a specific environment. At the molecular level different strategies exist that are used by cells and systems to respond and adapt to environmental changes such as changes of oxygen availability and temperature fluctuations. Environmental variations exceeding certain levels define “environmental stress.”

### Intrinsic developmental stress

An additional cause of physiological stress can be assigned to developmental events. As living organisms develop, they face a variety of challenges associated with morphogenesis and changes in inner chemistry. Indeed, rapid development of embryos causes massive internal changes to an organism as it grows and changes morphology. Different developmental events can cause different stressful conditions with some being harsher than others. Therefore adaptation to developmental stress is equally crucial for the survival of individuals and species.

### Aging

When development completes and organismal maturation is achieved, environmental stress is only one aspect of the physiological stress that challenges individuals. Aging constitutes another burden that living organisms have to cope with during their life. Even though aging has often been considered as the deteriorative result of different stresses, it can also be seen as an extra layer of stress throughout life due to the thermodynamic properties of biological materials that lead to the stochastic accumulation of molecular damage over time. The capacity of each organism to cope with aging and other stresses defines its longevity. Thus, functional decline through aging, which occurs even under physiologically perfect environmental conditions, may partly constitute the impact of entropy on organisms and reveals possible imperfections in homeostatic mechanisms.

### Role of neurons

**Figure 2:**
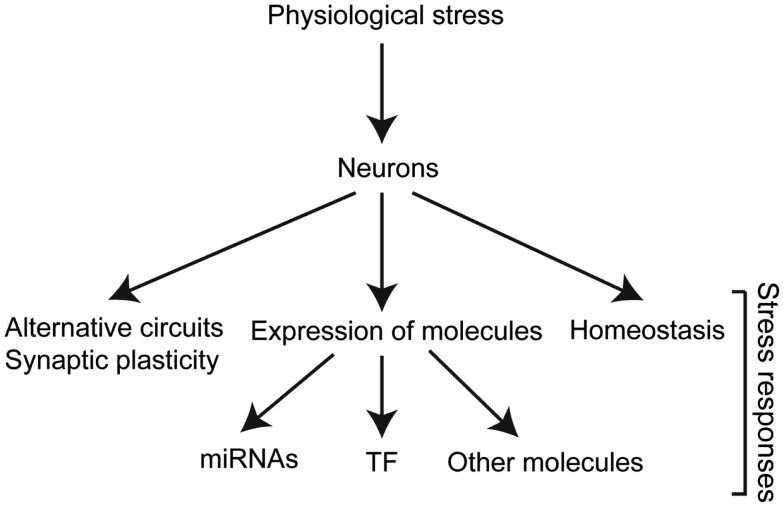
**Stress triggers biological responses in different levels of organization**. The different molecules involved dictate the changes required for adaptation, and therefore survival and reproduction. TF, transcription factors.

Research over the years has identified neurons as major players in stress responses. Neurons do not only receive and process information from the environment but they also have an important direct impact on different aspects of the stress response (Figure 2). In order for neurons to fulfill their roles in stress responses, specific molecules are temporally regulated in response to changes in internal or external conditions.

### Aim of the review

This review aims to provide examples that demonstrate the important role of neurons in physiological stress responses as well as the impact of physiological stress on neurons at the intercellular, cellular, and molecular level.

## Neuronal Responses

### Environmental stress and neuronal circuits

#### Hypoxia

Sensory neurons form cellular networks through which information from the environment is processed. In lower organisms these networks are relatively simple and stereotypic, and therefore they constitute attractive models to study the effects of physiological stress on neuronal information processing. From studies in *C. elegans*, in which the entire neuronal circuitry has been mapped (White et al., 1986), it has been found that stress can alter the processing of sensory information. In particular, under hypoxia, a latent circuit is engaged in the processing of gustatory information that is not normally used in normoxic conditions (Pocock and Hobert, 2010). In contrast, the aerotaxis neuronal circuit becomes simplified and less flexible after a hypoxic insult in the same organism (Chang and Bargmann, 2008). Interestingly, food-sensing and O_2_-sensing circuits in *C. elegans* can be altered by a natural gene variation underlining the specific adaptation of the neuronal circuits of different strains in diverse local environments (Cheung et al., 2005). Such plasticity described here alters behavioral responses that may provide the organism with advantages under stress. In higher organisms, where neuronal networks are extremely complex, there is a lack of information as to whether stress can alter the information flow through alternative neuronal networks in a similar way. However, in rats and other animals the so-called “cross phrenic phenomenon” has been observed where a latent respiratory motor pathway is activated by hypoxia, mediating faster recovery from spinal injury (Zhou et al., 2001). Together these examples show the functional plasticity of neuronal circuits and how they can alter information processing in response to environmental stress.

#### Preconditioning

The functionality of neuronal circuits under harsh environmental conditions can also be dependent on the prior exposure to different stresses (Robertson, 2004). Neurons that have been exposed previously to acute sub-lethal stress appear to retain a memory that allows them to survive and respond to higher doses of this stress than before their initial exposure. This phenomenon is called “preconditioning” or “neurohormesis” (Mattson and Cheng, 2006). Characteristic examples are the enhanced thermotolerance of neurons by prior heat shock in *Drosophila* (Karunanithi et al., 1999), in locusts (Dawson-Scully and Meldrum Robertson, 1998; Wu et al., 2001), and in *C. elegans* (Kourtis et al., 2012). Other examples include neuroprotection by prior hypoxic insult to subsequent ischemic conditions in mice (Miller et al., 2001), gerbils (Kitagawa et al., 1991), and in neuronal cell culture (Bruer et al., 1997). Recently it was shown that in piglets, ischemic preconditioning of a distant ischemic tolerant tissue protects the brain against ischemic injury, a phenomenon that is called “remote ischemic preconditioning,” and highlights the complexity of preconditioning mechanisms (Jensen et al., 2011). Interestingly, preconditioning of neurons can also be achieved by low doses of toxins naturally present in fruits and vegetables (Mattson and Cheng, 2006). In addition, exposure to a stress can induce tolerance to a different stress, a phenomenon that is called “cross-tolerance.” For example, in locusts, prior exposure to anoxia induces thermotolerance in neurons that control flight (Wu et al., 2002). In rats, prior exposure to high temperature enhances tolerance to spinal cord ischemia (Zhang et al., 2000). Finally, heat stress in murine cortical cell cultures enhance tolerance to combined oxygen and glucose deprivation (Snider et al., 1998). Preconditioning of neuronal circuits consist of an adaptive mechanism that uses prior experience to better confront hostile conditions. Moreover, stress cross-tolerance is a natural demonstration of the existence of common response mechanisms to different stresses such as high temperature and lack of oxygen.

#### Synaptic and neuronal network remodeling/plasticity

Another way that neurons respond to physiological stress is by altering their synaptic strength (functional plasticity) and/or connectivity pattern (structural plasticity) in a manner that promotes adaptation. In contrast to organisms like *C. elegans* where the neuronal connectivity appears to be stereotypical between individuals (White et al., 1986), the adult brain of higher animals exhibits a remarkable level of plasticity and the neuronal networks of certain regions can be altered under different conditions during and after their development (Pascual-Leone et al., 2011). Although this phenomenon has been mainly associated with learning and memory, it can also occur as a response to external stimuli and contributes to the homeostasis of the nervous system. For instance, sensory experience promotes the synaptic integration of new neurons into olfactory circuits in the mouse (Arenkiel et al., 2011). Moreover, chronic intermittent hypoxia alters the synaptic properties in a hub sensory circuit in rats (Kline et al., 2007), and in the peripheral chemoreceptor cells in the mollusk *Lymnaea stagnalis* synapses develop *in vitro* that exhibit a form of short-term synaptic plasticity in response to hypoxia (Bell et al., 2007). Finally, it is also characteristic that short-term plasticity of synapses is strongly dependent on temperature (Klyachko and Stevens, 2006). These examples show functional and structural plasticity of the nervous system in response to external stimuli.

### Environmental stress and individual neurons

#### Hypoxia

In addition to different responses that neurons exhibit upon particular stresses, they also exhibit various stress-related phenotypes. Because neurons are highly active cells, they require high amounts of oxygen in order to survive and function. Thus, hypoxia can have a tremendous impact on the physiology of animal brains. Limited oxygen availability during development (Lipton, 1999), at birth (Arpino et al., 2005; Gozzo et al., 2009), and later in life (Lipton, 1999) can cause irreversible damage to neuronal tissue. Hypoxia can also have an effect on axon outgrowth in a rat neuronal cell line (O’Driscoll and Gorman, 2005). In addition, specific *in vivo* developmental defects of individual neurons caused by hypoxia have been demonstrated in *C. elegans*. In this model, axon guidance and neuronal migration is defective in specific types of neurons under hypoxic conditions as a result of hypoxia inducible factor-1 hypoxia inducible factor (HIF)-1 stabilization (Pocock and Hobert, 2008). A recent study has also shown that similar defects are observed in the central nervous system of zebrafish via a similar pathway, providing strong evidence that this mechanism is conserved (Stevenson et al., 2012). Thus, hypoxia can have a pleiotropic impact on diverse neurons in different organisms.

#### Heat shock response

Increase in temperature (hyperthermia) is also known to affect many cell types. However, neurons are particularly susceptible to elevations in temperature, and organismal death under high temperature can be a result of neuronal malfunction before other cells fail (Robertson, 2004). High temperature mainly causes protein misfolding, which triggers cell autonomous and cell-non-autonomous responses (Ramirez et al., 1999). Certain sensory neurons are responsible for sensing the optimal temperature in freely moving animals as well as for the mediation of thermonociception (Clark et al., 2007; Liu et al., 2012). An interesting study in *C. elegans* has shown that such sensory neurons play a critical role in the cell-non-autonomous heat shock response of somatic cells (Prahlad et al., 2008). In addition, these neurons have also been shown to regulate a response to chronic stress caused by intracellular accumulation of misfolded proteins in remote somatic cells (Prahlad and Morimoto, 2011). Finally, the ciliated ASI chemosensory neurons in the same organism remotely regulate the proliferation vs. differentiation decision in gonads by secreting DAF-7/TGFβ when environmental conditions are favorable (Dalfo et al., 2012). In these ways, sensory neurons regulate systemic stress non-cell autonomously by integrating environmental inputs.

#### DNA damage response

Genotoxic factors such as UV and other electromagnetic radiations can also cause serious damage to neurons by damaging both their nuclear (Ide et al., 2000) and mitochondrial DNA (LeDoux et al., 2007). In contrast to other cell types that undergo cell cycle checkpoint arrest upon DNA damage, neurons seem to engage components of the cell cycle machinery in response to such insults (Park et al., 1997), as well as in response to other stresses such as ischemic hypoxia (Li et al., 1997; Timsit et al., 1999). Such cell cycle entry of neurons upon stress has been correlated with the apoptotic death after extensive DNA damage (Park et al., 1998; Herrup et al., 2004; Kruman et al., 2004). Moreover, entry of neurons into the cell cycle has also been correlated to neuronal cell death as an early disease related process (Herrup, 2012).

The majority of information on different DNA repair mechanisms available in cells comes from studies in non-neuronal mammalian cell systems. Despite the existence of diverse DNA repair pathways, base excision repair (BER) and nucleotide excision repair (NER) are the major mechanisms responsible for repairing oxidative-induced and UV-induced damage respectively, in both nuclear and mitochondrial DNA (Seeberg et al., 1995; Lagerwerf et al., 2011). Details of NER (Lagerwerf et al., 2011) and BER (Robertson et al., 2009) pathways have been recently reviewed. The gradual maturation of such repair mechanisms in neurons is shown by the fact that mature neurons appear to be more resistant to UV- and IR-induced DNA damage than their younger counterparts (Romero et al., 2003; Shirai et al., 2006). Neurons are also more resistant to IR-induced apoptosis compared to neuronal precursor cells (Kameyama and Inouye, 1994) and other cell types (Li et al., 1996). This highlights the importance of efficient DNA damage response (DDR) mechanisms to maintain mature post-mitotic cells, like neurons, which cannot be replenished (Romero et al., 2003). IR irradiation also affects multiple behavioral outputs of different species by affecting neurons (Sakashita et al., 2010 and references herein). Particularly in *C. elegans*, IR differentially affects neuron subtypes (Sakashita et al., 2010), which could also suggest that DNA repair efficiency varies between neuron types. Endogenous DNA damage also occurs in neurons. Neurons are highly active cells producing high levels of reactive oxygen species (ROS) that results in increased DNA damage in nuclear and mitochondrial DNA (LeDoux et al., 2007; Barzilai et al., 2008). Responses to endogenous DNA damage are crucial to enable correct neuronal development, and subsequent neuronal maintenance (LeDoux et al., 2007; Lee and McKinnon, 2007; Barzilai et al., 2008). The importance of the DDR in the absence of external mutagenic factors is also supported by genetic conditions in humans that cause developmental defects (O’Driscoll and Jeggo, 2006; Barzilai et al., 2008). Finally, neurons can remotely protect other tissues from insults such as ionizing irradiation. A striking example comes from *C. elegans* where DNA damage-induced apoptosis in the worm gonad is negatively regulated by a pathway involving HIF-1. Specifically, HIF-1 acts in ASJ amphid sensory neurons to upregulate the tyrosinase family member TYR-2. TYR-2 is subsequently secreted from these neurons and downregulates CEP-1, the homolog of p53, in gonads thereby suppressing radiation-induced apoptosis (Sendoel et al., 2010). It is thus clear that neurons are especially susceptible to both exogenous and endogenous genotoxic reagents and cells can react in both a cell autonomous and non-cell autonomous manner to promote survival.

### Intrinsic developmental stress and the nervous system

Stressful conditions in the internal somatic microenvironment of organisms can be caused by development (Simon and Keith, 2008). Even though development has been recognized as an additional layer of stress, there is limited knowledge as to how it influences the nervous system. Developmental stress is unique in its nature by being stereotypical during embryonic and postembryonic life. Therefore, the responses to this stress have to be embedded into neurons and in some cases may serve as a necessary part of their development. The most well studied stressor during development is intrinsic hypoxia. In the developing embryo, hypoxic regions naturally occur as a result of limited O_2_ distribution (Simon and Keith, 2008) where the major hypoxia regulator, HIF-1 plays extensive roles (Dunwoodie, 2009). Interestingly, low oxygen levels during development are important for differentiation of many cells and tissues (Morriss and New, 1979; Maltepe and Simon, 1998; Simon et al., 2002) and studies in neuronal cell culture suggest that this might also be true for neurons (Morrison et al., 2000; Studer et al., 2000). Therefore, it seems that development uses an inherent stressful condition, such as embryonic hypoxia, as a signal to form various structures. Thus, it has been suggested that O_2_ functions as a developmental morphogen (Simon and Keith, 2008). In addition, the observed hypoxic tolerance of developmentally immature neurons compared to mature neurons is indicative of the adaptation of neurons to developmentally derived hypoxia (Bickler and Buck, 1998).

Neurons can also help the embryo to overcome developmental stress at the behavioral level. In the pond snail *Helisoma trivolvis* the cilia-driven rotational behavior of early embryos facilitates gas exchange with the surrounding liquid and is regulated by a pair of serotonergic sensory-motor cells that sense oxygen levels (Kuang and Goldberg, 2001; Kuang et al., 2002). This embryonic behavior has also been observed in other species such as in the pond snail *Lymnaea stagnalis* (Byrne et al., 2009). Other stressors can also be present during development. For example, oxidative stress is produced as a result of routine adult neurogenesis (Walton et al., 2012). The developing brain has adopted a variety of different defenses against developmentally derived oxidative stress (Ikonomidou and Kaindl, 2011), such as differential expression of antioxidant systems during brain development (Aspberg and Tottmar, 1992). Finally, the nervous system is subjected to mechanical stress via movement and from the increasing mass of the brain during development (Van Essen, 1997; Benard and Hobert, 2009). This developmentally derived mechanical stress results in a variety of neuronal responses at different levels (Benard and Hobert, 2009). The above examples describe the existence of developmental stress as an important aspect of development and stress biology. Neurons not only adapt to stress but they also *require* specific stressors for correct development to occur.

### Aging and the nervous system

Aging is perceived as the deteriorative effect of time on different structures of living organisms and many theories have been developed to date to explain how aging evolved in different organisms (Kirkwood and Austad, 2000). Early studies showed a relation between sensory neurons and longevity (Apfeld and Kenyon, 1999). Different types of sensory neurons have been found to regulate *C. elegans* lifespan in both positive and negative manner, emphasizing the underlying complexity of such regulation (Alcedo and Kenyon, 2004; Bishop and Guarente, 2007; Lee and Kenyon, 2009; Shen et al., 2010a,b). Neurons can impact on longevity in a non-cell autonomous manner. For example, upon neuronal specific mitochondrial stress a cue from the nervous system in *C. elegans* induces the mitochondria-specific unfolded protein response in intestinal cells, thus increasing the lifespan of the animals (Durieux et al., 2011). In addition, neuroprotection plays a critical role in longevity and aging (Murakami, 2007) and the regulation of aging via neurons appears to be conserved in *Drosophila* (Parkes et al., 1999; Libert et al., 2007). This association between sensory neurons and longevity shows the non-cell autonomous impact that environmental cues have in organismal aging.

Aging is also known to impact on individual neurons. Beyond pathological conditions, e.g., Alzheimer’s disease and Parkinson disease, which have been well documented over the years (Yankner et al., 2008; Hung et al., 2010), neurons undergo important morphological and functional changes during normal aging. Invertebrate models have been extensively used and have revealed a number of age-related neuronal events. For example, *C. elegans* touch receptor and cholinergic neurons display age-dependent morphological defects such as cytoskeletal disorganization, axon beading, and defasciculation (Pan et al., 2011). Moreover, unexpected ectopic branching of neurites has been recently observed in *C. elegans* neurons as a result of aging (Tank et al., 2011). Although this branching was linked to impaired mechanosensory perception and decreased mobility, it seems to be regulated independently of organismal lifespan (Tank et al., 2011). Based on this notion, we could hypothesize that neuronal branching plays a survival role in aged worms in the wild, but not under laboratory conditions and that it is not just a result of aging. This hypothesis would also explain why the branching is regulated by age-related pathways, such as the Jun kinase and the insulin/IGF-1 pathways (Tank et al., 2011). As these pathways are known to affect neuronal plasticity across species (Sherrin et al., 2011; Antoniou and Borsello, 2012; Fernandez and Torres-Aleman, 2012), it is not surprising that they also affect *C. elegans* neurite branching. Finally, the protection of neurons from aging relies heavily on the general lifestyle of individuals, e.g., diet and exercise, which highlights further the complexity of aging mechanisms (Stranahan and Mattson, 2012).

In higher organisms normal aging influences different parts of the brain at different rates (Woodruff-Pak et al., 2010) and impacts on synaptic connectivity of specific areas of the brain. For example, in the olfactory bulb in mice, the synaptic density of olfactory sensory neurons decreases with age in the glomerular layer but not the external plexiform layer (Richard et al., 2010). Interestingly, other neuronal characteristics, as well as neuronal populations are unaffected in the same region during aging (Richard et al., 2010). Such a selective effect of aging on different synaptic populations is not well understood. However, synaptic dysfunction during aging is conserved and it has been observed in different monkey species (Page et al., 2002; Duan et al., 2003) and in rats, where a study estimated a 27% decrease in axodendritic synapse number in the middle molecular layer of the dentate gyrus in 25 month old individuals compared to those of 3 months old (Bondareff and Geinisman, 1976). The relation between aging-related neuronal phenotypes and cognitive impairment is also not clear. An interesting study in *Drosophila* has recently made a functional connection between memory loss and impairment in specific neurons during normal aging (Tonoki and Davis, 2012). The authors were also able to retrieve lost memories due to aging by prior stimulation of these neurons (Tonoki and Davis, 2012). Despite the high number of different neuronal defects associated with aging, relatively few neurons die during normal aging (Herndon et al., 2002; Burke and Barnes, 2006). However, a recent study has shown that there is a significant and specific loss of hyperploidic neurons (neurons that contain more than a diploid number of chromosomes) with aging in the cerebral cortex of normal human brain (Fischer et al., 2012). Such specific neuronal loss is yet to show whether it contributes to age-related impairments. In general, neuronal decline rather than neuronal loss seems to be responsible for the negative manifestations of normal aging such as memory loss.

The cause of neuronal changes during aging is not completely understood. A common suspect appears to be increased oxidative stress and is in accordance with the “oxidative stress theory of aging” (Gerschman et al., 1954; Harman, 1956; Cadet, 1988). Oxidative stress is the negative impact of ROS on different aspects of cellular function and can lead to molecular defects such as DNA and mitochondria damage. It is characteristic that genes associated with stress responses and DNA repair are upregulated in the aging human brain (Lu et al., 2004). In normal aging, generation of ROS is elevated due to alterations in neuronal calcium handling and changes in lipid peroxidation (Stranahan and Mattson, 2012). These molecular events can lead to suppression of adult neurogenesis and implementation of alternative plasticity mechanisms to compensate for the damaged tissue (Stranahan and Mattson, 2012). Neurons are particularly vulnerable to oxidative stress, which can lead to neuronal cell death associated with many age-related neurodegenerative diseases (Coyle and Puttfarcken, 1993; Andersen, 2004). Nevertheless, recent studies question the role of oxidative stress in aging (Doonan et al., 2008; Yen et al., 2009; Van Raamsdonk and Hekimi, 2010, 2012; Hekimi et al., 2011). In addition, although it is not known whether any neurons are involved, mild elevations of ROS can be beneficial for a longer lifespan in *C. elegans* (Lee et al., 2010).

Thus, in general, aging is actively regulated by the nervous system and aging in turn influences neuronal properties. The nature of aging as a stressor appears to go beyond the result of environmental stress and can extend to an inevitable decline of repair mechanisms possibly due to physical entropy.

### Neuronal homeostasis

Much of the ability of nervous system to fulfill its role relies on its homeostatic capability. This process is known as “homeostasis” and has been defined as “the maintenance of the constancy of the internal environment” (Turrigiano and Nelson, 2004). Regardless of changes in their environment, the structural and functional integrity of neurons must be conserved throughout life. When homeostatic mechanisms go awry, neurons decline and become unable to respond to external disturbances. This leads to many age-related neurodegenerative diseases, as well as other neuronal defects (Ramocki and Zoghbi, 2008). During development, neurons and neuronal networks put in place a variety of regulatory mechanisms in order to maintain their function despite changes in their microenvironment (Turrigiano and Nelson, 2004). The nervous system is also subjected to a variety of physical stresses throughout life. For example, the addition of new cells into adult neuronal circuits during neurogenesis tends to destabilize the functionality of circuits and dictates homeostatic adaptations in different levels (Meltzer et al., 2005). Neurons can also be challenged from physical body movements, muscle contractions, and injury, all of which have to be confronted by homeostatic mechanisms (Benard and Hobert, 2009). To this end neurons utilize extracellular matrix components, cell adhesion molecules and cytoskeletal proteins to maintain architectural integrity (Benard and Hobert, 2009). Thus, homeostatic mechanisms seem to act in the opposite direction to aging and disease in a complex and dynamic manner.

## Key Molecules Involved

The regulation of protein expression under stress is complex and includes mechanisms such as epigenetic gene regulation, transcriptional regulation, and post-transcriptional regulation. Many molecules have been reported in many different species to mediate the cellular responses to stress. However, only a fraction of them have specific roles in neurons during these responses (Table 1). We summarize here studies for some key molecules involved in neuronal stress responses.

**Table 1:** **Molecules involved in neuronal responses to different stresses in various organisms**.

Molecule type		Name	Organism	Role	Reference
**Transcription factors**		bZIP transcription factors and related targets	*M. mu*^1^	ER stress response, hypoxia-induced neuronal death	Halterman et al. (2010)
		Activator protein-1 (AP1)	*H. sa*^2^, *M. un*^3^	Hypoxia	McGahan et al. (1998), Domanska-Janik et al. (1999)
		P53	*H. sa*	DNA damage response, oxidative stress	Culmsee and Mattson (2005)
		RORα (retinoid-related orphan receptor-α)	*M. mu*	Hypoxia	Jolly et al. (2011)
		Nuclear factor kappa-light-chain-enhancer of activated B cells (NFκB)	*H. sa*	Various stresses including hypoxia	Qiu et al. (2001), Massa et al. (2006)
		SKN-1	*C. el*^4^	Oxidative stress, longevity	An and Blackwell (2003), Bishop and Guarente (2007)
**Phosphatidylinositol 3-kinase-related kinases (PIKKs)**		ATPase associated diverse cellular activities (AAA+) proteins RUVBL1 and RUVBL2 (RUVBL1/2)	*H. sa*	DNA damage response	Izumi et al. (2012)
		ATM protein kinase	*H. sa*	DNA damage response, synaptic plasticity	Abraham (2001), Li et al. (2009a), Tian et al. (2009)
		Ataxia- and Rad3-related neuron (ATR)	*R no*^5^	DNA damage response	Ye and Blain (2010)
		DNA-dependent protein kinase catalytic subunit (DNA-PKcs)	*M mu*	DNA damage response	Chechlacz et al. (2001)
		Cyclin dependent kinase 5 (Cdk5)/p35 complex	*M. mu*, *H. sa*	Hypoxia. DNA damage response	Antoniou et al. (2011), Zhu et al. (2011)
**Signaling molecules**		TNF-α, NRF2, and CREB	*R. no*	Preconditioning, oxidative stress	Shih et al. (2005), Saha et al. (2009)
		Tumor suppressor warts/lats (Wts)	*D. me*^6^	Dendritic maintenance	Emoto et al. (2006)
		Neurotrophins	*H. sa*	Neuronal maintenance	Henderson (1996)
		Unfolded protein response (UPR) system	*H. sa*	ER stress	Sammeta and McClintock (2010), Hoozemans and Scheper (2012)
		AMP-activated protein kinase (AMPK)	*H. sa*, *C. el*	Axogenesis during metabolic stress, neuronal plasticity, longevity	Schulz et al. (2007), Potter et al. (2010), Williams et al. (2011)
**Extracellular**		DGN-1, ANC-1	*C. el*	Neuronal maintenance	Johnson and Kramer (2012)
**components**		DIG-1	*C. el*	Neuronal maintenance	Benard et al. (2006), Burket et al. (2006)
		Collagen VI	*M. mu*	Neuroprotection after UV exposure	Cheng et al. (2011)
**Histone deacetylate**		Sirtuin (silent mating type information regulation 2 homolog)	*H. sa*, *C. el*	Oxidative stress	Gan and Mucke (2008)
**Ion channel**		Thermo transient receptor potential (thermoTRP)	*H. sa*	Thermo-avoidance	Dhaka et al. (2006)

*^1^*Mus musculus*, ^2^*Homo sapiens*, ^3^*Meriones unguiculatus*, ^4^*Caenorhabditis elegans*, ^5^*Rattus norvegicus*, ^6^*Drosophila melanogaster**.

### Transcription factors

#### Hypoxia inducible factors

**Figure 3:**
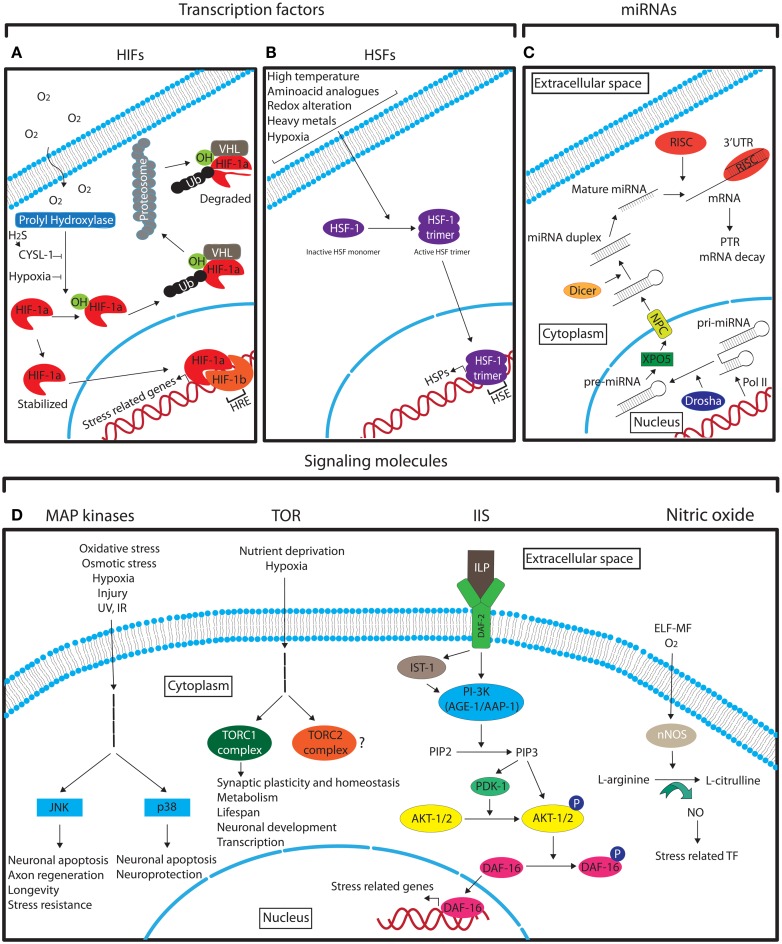
**Schematic representation of the different main molecular pathways that are involved in neuronal stress response**. See the text for detailed description. **(A)** The role of HIFs in hypoxia response. **(B)** The role of HSF-1 in stress response. **(C)** The biogenesis of miRNAs. **(D)** The main signaling pathways that mediate the neuronal response to different stresses. Legend: arrows indicate the pathway flow and/or the positive effect of an element onto another. Blunted arrows indicate inhibition. Question mark denotes lack of information. Ub, ubiquitin; TF, transcription factors.

Hypoxia inducible factors are the key modulators of hypoxic stress responses. They function as heterodimers consisting of an oxygen regulated α and a stable β subunit. The HIF heterodimer binds to the promoter of target genes via hypoxia response elements (HREs) with the consensus sequence G/ACGTG (Figure 3A; Majmundar et al., 2010). These target genes regulate a vast array of processes that enable cellular adaptation to hypoxia. HIFα is primarily regulated by oxygen-dependent prolyl hydroxylase-domain enzymes (PHDs) that lead to its degradation via the von Hippel–Lindau tumor suppressor protein (VHL) under normoxia (Epstein et al., 2001). In hypoxic conditions PHD activity is diminished and HIFα is stabilized (Figure 3A; Epstein et al., 2001; Majmundar et al., 2010). A recent study in *C. elegans* has also implicated the homolog of sulfhydrylases/cysteine (CYSL-1) in stabilizing HIF-1α in neurons via EGL-9, the worm PHD homolog, as a response to hypoxia-derived intracellular hydrogen sulfide (H_2_S; Figure 3A; Ma et al., 2012).

Various studies have shown a specific role of HIF-1 in neurons. Depletion of HIF-1α in the mouse brain and in neuronal cell cultures causes increased cell damage and lower survival rate after cerebral ischemia (Baranova et al., 2007). In rat cortical neurons, HIF-1α appears to play a protective role in early steps of responses to mild hypoxia (Lopez-Hernandez et al., 2012). HIF-1α has recently been shown to regulate prion protein expression in hippocampal neuronal cells to protect from cell damage (Jeong et al., 2012). Moreover, it is not clear whether HIF-1α plays a central role in hypoxic preconditioning as different studies argue for (Grimm et al., 2005; Shao et al., 2007; Ara et al., 2011) and against (Li et al., 2011) such a role. Although HIF-1 retains neuroprotective and anti-apoptotic properties, there is experimental evidence that point to a deteriorative impact of stabilized HIF-1α on neuronal tissue. For example, HIF-1 stabilization causes axon guidance and neuronal migration defects in *C. elegans* (Pocock and Hobert, 2008) and promotes neurodegeneration in neonatal rat brain (Jiang et al., 2012). In addition, HIF-1 in *C. elegans* negatively regulates lifespan extension by dietary restriction, acting in the serotonergic neurons ADF and NSM (Chen et al., 2009). However, studies from other groups report that in the same organism HIF-1 promotes longevity (Mehta et al., 2009; Zhang et al., 2009; Lee et al., 2010). This discrepancy was addressed in two more recent studies where it was shown that HIF-1 regulates longevity in a temperature-dependent manner (Hwang and Lee, 2011; Leiser et al., 2011). Thus, it is possible that the role of HIF-1 in longevity is context-dependent and may involve different neurons or other cells under the different conditions. While *C. elegans* neurons have also been recently found to sense and respond to hypoxia in a HIF-independent manner (Park et al., 2012), HIFs appear to be the main factors mediating neuronal responses to hypoxia through the regulation of many downstream effectors. This is despite the fact that neuroprotective properties of HIF may cause side effects on other aspects of neuronal physiology.

#### Heat shock factors and heat shock proteins

Heat shock factors (HSFs) are stress-inducible transcription factors that upon induction positively regulate the expression of heat shock proteins (HSPs) through direct binding to their promoters containing heat shock elements (HSEs; NGAAN; Shamovsky and Nudler, 2008). The master regulator of heat shock response is the HSF-1, which under normal conditions is in a monomeric inactive form (Shamovsky and Nudler, 2008). Upon different stress conditions HSF-1 forms an active trimer that enters the nucleus in order to activate HSPs (Figure 3B; Shamovsky and Nudler, 2008). The regulation of HSPs by HSFs is highly conserved from yeast to mammals (Liu et al., 1997). HSPs function as molecular chaperones to facilitate the proper folding of other cellular proteins. Protein misfolding can originate under normal cellular conditions, and under different stresses. It has also been shown that over-excitation of motor neurons can cause protein misfolding in post-synaptic muscle cells in *C. elegans* (Garcia et al., 2007). The induction of HSFs in neurons and in other cell types is not only stimulated by hyperthermia as its name implies, but also by other stresses, such as, hypoxia, alterations in the intracellular redox environment, and exposure to heavy metals and amino acid analogs (Morimoto et al., 1997). HSPs have also been shown to be upregulated in ischemic preconditioning (Liu et al., 1993; Kato et al., 1995). The neuroprotective properties of HSF-1, the principal regulator of heat shock response in *C. elegans*, have been demonstrated in two recent studies where upregulation of HSF-1 suppresses the defective neuronal phenotypes of a Machado–Joseph disease mutant model (Teixeira-Castro et al., 2011) and HSF-1 and the small heat shock protein HSP-16.1 mediate cytoprotection by heat preconditioning (Kourtis et al., 2012). In addition to HSPs, HSFs can induce the transcription of other proteins with various functions (Akerfelt et al., 2010). Some of them have neuroprotective functions under stress. For example, under heat shock in *Drosophila*, HSFs induce the expression of the NAD synthesis enzyme, nicotinamide mononucleotide adenylyltransferase (NMNAT), which is critical for neuronal maintenance under stress (Ali et al., 2011).

The expression of HSF-1 in neurons appears to be strictly controlled (Dirks et al., 2010) and the increased susceptibility of neurons to heat shock treatment is associated with the delayed onset of HSF-1 expression (Batulan et al., 2003; Kern et al., 2010). However, HSPs may have a more neuronal specific role under normal conditions, as some HSPs are constitutively more highly expressed in neurons than other cells (Chen and Brown, 2007). The beneficial role of the expression of HSPs was recently recognized (Rordorf et al., 1991) and can be induced by chemical compounds serving as medical drugs against different degenerative diseases (Katsuno et al., 2005; Chow and Brown, 2007). Many HSFs and HSPs are also up- and down-regulated during normal development of different species (Akerfelt et al., 2010), which may suggest a protective role for these proteins with regards to intrinsic developmental stress. Moreover, HSPs are related to normal and abnormal embryonic development (Evans et al., 2005; Brown et al., 2007) and the expression of HSPs has shown to be phase- and tissue-specific (Loones et al., 1997; Masuda et al., 1998). HSFs and HSPs are also implicated in aging. Hsp22, when over-expressed in motor neurons was shown to increase the lifespan of *Drosophila* by 30% (Morrow et al., 2004) and the flies maintained their locomotory activity longer and were more resistant to oxidative stress and hyperthermia (Morrow et al., 2004). In *C. elegans* HSF-1 promotes longevity by acting in neurons and other tissues (Lithgow et al., 1995; Hsu et al., 2003; Morley and Morimoto, 2004). Finally, the heat shock response of AFD and AIY thermosensory neurons in *C. elegans* involves upregulation of Hsp70 protein (Prahlad et al., 2008). It is thus apparent that HSFs and HSPs are involved in the core of the response mechanisms against many different stresses with particular importance in neuronal cell function.

### Signaling molecules

#### Mitogen-activated protein kinases

Mitogen-activated protein (MAP) kinases are essential signal transduction molecules that mediate the response to environmental cues in virtually all cell types and play key roles in cellular functions such as differentiation, cell survival, and apoptosis (Gehart et al., 2010). These and other general roles of certain MAP kinases in neurons was demonstrated several years ago (Fukunaga and Miyamoto, 1998). Sub-families of MAPKs have been recognized to date such as the extracellular signal-regulated kinases (ERK1/2), ERK5 (also known as BMK1 or MAPK7), the Jun amino-terminal kinases (JNK) 1–3, and the p38 kinases (p38α, β, γ, and δ; Gehart et al., 2010). The exact content and features of the different MAPK pathways have been described elsewhere (Dhillon et al., 2007). However, the general scheme of MAPK signaling follows the sequence “stimulus – G-protein – MAPKKK – MAPKK – MAPK- final response” (Dhillon et al., 2007). The stress-activated MAPK pathways are essentially the JNK and the p38 kinases (Figure 3D; Dhillon et al., 2007).

The JNK pathway can be induced by oxidative and osmotic stress, UV radiation, and other DNA-damaging agents to modulate the Activator protein-1 (AP1) and other transcription factors, such as HSF-1 and HIF-1 (Park and Liu, 2001; Antoniou and Borsello, 2012) in order to regulate the cellular stress responses. Neuronal apoptosis after stress is positively regulated by the JNK pathway (Weston and Davis, 2002; Biteau et al., 2011) and JNK/MAPK signaling is also involved in axon regeneration after injury in *C. elegans* (Hammarlund et al., 2009; Yan et al., 2009; Li et al., 2012). Moreover, the JNK pathway in neurons promotes organismal longevity by activating neuroprotective mechanisms in *Drosophila* (Lee et al., 2009; Biteau et al., 2011; Takahama et al., 2012), and in *C. elegans* (Oh et al., 2005). Finally, the JNK pathway can act by modulating FOXO transcription factors and antagonizes Insulin/IGF-1 factors to regulate different aspects of stress resistance and aging (Wang et al., 2005; Neumann-Haefelin et al., 2008).

p38 kinase pathway can be induced by stresses such as hypoxia, oxidative stress, IR, and UV irradiation and it is mainly implicated in the induction of neuronal apoptosis under different stresses (Horstmann et al., 1998; Namgung and Xia, 2000; Choi et al., 2004; Guo and Bhat, 2007). The p38 pathway also appears to play a role in neuroprotection against ischemia after isoflurane preconditioning (Zheng and Zuo, 2004). However, the p38 pathway has other roles in neurons beyond stress responses (Takeda and Ichijo, 2002).

#### Target of rapamycin

Target of rapamycin (TOR) is an evolutionary conserved serine/threonine kinase important predominantly in regulating cell growth and proliferation, with implications in many different aspects of development, aging, and disease (Wullschleger et al., 2006). TOR functions as a sensor of extracellular signals, including stressors such as hypoxia and nutrient deprivation, and is found in two functionally distinct complexes, namely TORC1 and TORC2 (Figure 3D). Inhibition of TOR signaling increases lifespan in many organisms, including mice and *Drosophila*, partly by reducing mRNA translation (Harrison et al., 2009; Bjedov et al., 2010). Even though mammalian TOR is ubiquitously expressed, it plays an extensive role in neuronal development and plasticity (Jaworski and Sheng, 2006; Chong et al., 2010; Hoeffer and Klann, 2010). TOR has also been recently found to be important for synaptic homeostasis in *Drosophila* (Penney et al., 2012) and for synaptic plasticity after ischemia in rats (Ghiglieri et al., 2010). mTOR may also be involved in the regulation of HIF-1alpha in the developing rat brain with hypoxia-ischemia (Chen et al., 2012). However, the role of TOR signaling in mediating stress responses in neurons has not been fully elucidated.

##### Insulin/IGF-1 signaling

The insulin/IGF-1 pathway is a very well characterized pathway that regulates aging and longevity in many different species (van Heemst et al., 2005). The complexity of Insulin/IGF-1 signaling (IIS) pathway has been greatly increased during evolution (van Heemst et al., 2005). In the nematode *C. elegans*, where the involvement of this pathway in aging was first discovered and extensively studied, the insulin-like growth factor receptor DAF-2 is activated by insulin-like peptides (ILP; 40 encoded in worm genome) that are primarily expressed in neurons (Pierce et al., 2001; Li et al., 2003; Husson et al., 2007; Cornils et al., 2011). After ligand binding, the signal is transduced directly or via the insulin receptor substrate homolog protein-1 (IST-1) to the phosphatidylinositol 3-kinase (PI-3K) consisting of the catalytic subunit AGE-1 (aging alteration-1) and the regulatory subunit AAP-1 (phosphoinositide kinase AdAPter subunit), which converts phosphatidylinositol 4,5-bisphosphate (PIP_2_) into phosphatidylinositol 3,4,5-trisphosphate (PIP_3_; van Heemst, 2010). PIP_3_ activates the 3-phosphoinositide-dependent protein kinase-1 (PDK1) and the protein kinases B (known as AKT-1/2), leading to the phosphorylation of DAF-16, a homolog of the mammalian FoxO family of transcription factors (van Heemst, 2010). Phosphorylated DAF-16 is retained in the cytoplasm whereas unphosphorylated DAF-16 enters the nucleus to regulate a battery of stress response genes (Figure 3D; Lin et al., 2001; Lee et al., 2003a; Murphy et al., 2003).

Although the insulin receptor was found expressed in neuronal tissue (Havrankova et al., 1978; Unger et al., 1989), and its distribution appears to be enriched in particular brain areas (Schulingkamp et al., 2000), its role in neurons was not clear. As neurons can take up glucose without the involvement of insulin or insulin receptor, these cells were believed to be “insulin insensitive.” Cline and colleagues provided the first *in vivo* evidence that the insulin pathway regulates neuronal circuit function and synaptic maintenance in the central nervous system of *Xenopus* tadpoles (Chiu et al., 2008). Previous studies had also demonstrated the role of insulin signaling in the regulation of lifespan in *C. elegans* neurons (Kenyon et al., 1993; Kimura et al., 1997; Apfeld and Kenyon, 1998; Wolkow et al., 2000), in *Drosophila* neuroendocrine cells (Tatar et al., 2001), and in the mammalian brain (Kappeler et al., 2008). A number of other studies have identified roles of the neuronal insulin pathway in energy homeostasis (Konner et al., 2011; Freude et al., 2012), synaptic plasticity (Oda et al., 2011; Costello et al., 2012), and neuronal apoptosis following hypoxic insult (Liu et al., 2011). Interestingly, IIS has also been shown to regulate salt chemotaxis learning in *C. elegans* (Tomioka et al., 2006). The insulin pathway can also act on neurons non-cell autonomously to regulate neuronal aging (Pan et al., 2011) and has also been shown to act in neurons to suppress organismal survival under hypoxia (Scott et al., 2002). In general, the insulin pathway has extended roles in neuronal tissues of many organisms that can span from the regulation of aging to hypoxia sensitivity.

#### Nitric oxide

Nitric oxide (NO) is a free radical important for cell signaling with a number of physiological roles such as synaptic plasticity and neurotransmission. The enzyme responsible for NO production in neurons is the neuronal nitric oxide synthase (nNOS) that mediates the generation of l-citrulline from l-arginine (Figure 3D; Zhou and Zhu, 2009). nNOS responds to oxygen levels and is upregulated by hypoxia in different neurons (AbuSoud et al., 1996; Prabhakar et al., 1996). NO regulates a variety of stress-related transcription factors such as HIF-1 and NF-κB (Contestabile, 2008; Keswani et al., 2011) and can lead to neuronal death under different stress conditions (Brown, 2010). Interestingly, NO in neurons can also be induced by extremely low frequency magnetic fields (ELF-MF) in the rat brain, a usual stressor emanated from electrical devices (Cho et al., 2012). Therefore, NO appears to be an important inorganic molecule for neuronal stress responses.

### MicroRNAs

MicroRNAs (miRNAs) are small non-coding RNA molecules with a length of approximately 22 nucleotides that act as post-transcriptional regulators of gene expression (Ebert and Sharp, 2012). They have the ability to fine-tune expression to ensure stability under sudden external or internal perturbations or, if needed, enforce a new gene expression program that enables an organism to tolerate a new environment (Ebert and Sharp, 2012). miRNAs are transcribed, mostly by RNA polymerase II (Pol II), as capped and polyadenylated primary miRNAs (pri-miRNAs) that fold in extended hairpin structures. The pri-miRNAs are cleaved in the nucleus by the RNase III enzyme Drosha, creating the shorter precursor miRNA (pre-miRNA; Lee et al., 2003b). The pre-miRNA is transported by exportin-5 (XPO5) via the nucleopore complex (NPC; Yi et al., 2003) out of the nucleus (Lee et al., 2011) where is further processed by the RNase II enzyme, Dicer, and is incorporated into an Argonaute-containing RNA-induced silencing complex (RISC; Lee et al., 2002). In the RISC complex, the miRNA associates with a specific target mRNA by imperfectly base-pairing with its 3′ UTR and mediates post-transcriptional repression (PTR) or decay of specific mRNA targets (Figure 3C; Lee et al., 2002, 2003b, 2011; Pasquinelli, 2012). Robustness in systems that control cell fate, developmental transitions, and stress responses is required under changing conditions as fluctuations in the internal or external environment can be fatal for an organism. miRNAs can generate rapid and reversible responses and, in this way, are ideal molecules for mediating stress responses. As deletion of individual miRNAs (Miska et al., 2007) or whole miRNA families in *C. elegans* (Alvarez-Saavedra and Horvitz, 2010) have little to no effect on viability and development and as most of the miRNA knock-out mice do not show any gross phenotypes (Park et al., 2010), it is believed that the main function of miRNAs may be to buffer gene expression when an organism is challenged under stressful conditions. Supporting this idea Zhang and colleagues showed that deletion of *mir-71* impaired the long-term survival of nematodes during starvation-induced L1 diapause (Zhang et al., 2011b). In addition, miR-7 was shown to be essential for the maintenance of regulatory stability under temperature stress during development of a *Drosophila* sensory organ (Li et al., 2009b). miRNAs are abundantly expressed in the nervous system and a relation between miRNAs and neuronal responses to stress has been demonstrated in different model systems. Most recently, *mir-71* has also been associated with an increase in lifespan in *C. elegans* (Boulias and Horvitz, 2012). This study showed that expression of *mir-71* in neurons alone was sufficient to promote germline-mediated longevity and proposed a model in which *mir-71* mediates lifespan-extending signals through the DAF-16/FOXO transcription factor in the nervous system. The direct mechanism by which the internal stress is sensed by the neurons is not clear; however the function of miR-71 was shown to be partly dependent on expression of TCER-1 (Boulias and Horvitz, 2012), a transcription elongation factor shown to promote the transcriptional activity of DAF-16 in the intestine (Ghazi et al., 2009). Systemic stress can also be triggered by alcohol and neuronal adaptation to this stress was shown to cause a rapid increase in miR-9 expression in neurons (Pietrzykowski et al., 2008). This led to transcription of a voltage-activated potassium channel isoform associated with an increase in alcohol tolerance. This process may represent a general mechanism of neuronal adaptation to alcohol and suggests that miR-9 has an important role in neuronal plasticity (Pietrzykowski et al., 2008). A study in primary rat hippocampal neuronal cells has also shown that under hypoxia, the miR-130 family is highly expressed (Saito et al., 2011). In particular, miR-130a appears to decrease DDX6 protein levels, the normal function of which is to restrict HIF 1α (HIF-1α) mRNA to P-bodies of hippocampus neuronal cells of mice (Saito et al., 2011). Under hypoxia, HIF-1α mRNA is released from these foci and the protein can regulate oxygen homeostasis (Saito et al., 2011). The expression of miRNAs in response to stress has also been demonstrated to be cell specific. In a primary cell culture-based assay, astrocytes and neurons were subjected to oxygen-glucose deprivation to mimic ischemia, which is an essential feature of traumatic brain injuries (Ziu et al., 2011). In this model, different panels of miRNAs were upregulated in the two cell types, indicating that different neurons utilize different biochemical pathways to respond to physiological stress (Ziu et al., 2011). Together these studies suggest that miRNAs are implicated in neuronal stress responses. However, further research is expected to address more questions as to how miRNAs regulate neuronal responses to stress, and determine their impact on organismal homeostasis.

## Discussion

The physical world is built around principles that dictate the usage and management of energy. These principles are all connected by the basic laws of physics, and obey the same basic restrictions and limitations. We can think of living organisms as biological engines using their structures to utilize energy in order to survive and reproduce in a certain environment. The negative biological impact of stress on living systems relies on the inability of the latter to function and continue utilizing energy under certain conditions. This may either reflect the limitations of certain basic principles when, for example, no life can thrive at a temperature of absolute zero (−273°C), or a lack of adaptation of a particular organism to a given environment when, for example, an elephant cannot survive in Antarctica, while a polar bear cannot survive in the African savannah. Apart from such extreme mismatches between life and environmental conditions, there are milder ones that define stress as we discuss here. Stressful conditions often differ between organisms as different species have adapted to different environments. The extent to which each species has adopted defense mechanisms against stressors may reflect the extent of the presence of the relevant conditions during evolution.

Intrinsic developmental stress and aging can also count as selective factors for genes that give a survival advantage in the course of evolution, in the same manner as external environmental stress does. Stress derived from development may have led organisms to adopt particular developmental programs that lead to the final structures; and aging may have selected molecular pathways that lead different organisms to achieve a health span that fits their ecological role. Considering the cellular properties of neurons and their documented implication in stress responses, the nervous system could constitute a “hot-spot” for the evolutionary adaptation of organisms to different stresses. In this sense, stress can be seen as a driving force of biodiversity in the long term that may have been particularly applied on the different levels of neuronal organization to select for the variety of the adaptation mechanisms to stress we presented here. However, the role of neurons on aging of higher organisms is not yet well understood. Much of our knowledge in this field comes from invertebrate models where it is characteristic that long-lived animal mutants show tolerance in different stresses (Kourtis and Tavernarakis, 2011). Yet there is clearly an additional level of active regulation of aging and longevity by neurons that is beyond a simple resistance to environmental stress.

During the life of an organism, stress conditions subject a burden that needs to be confronted and overcome in order to survive and reproduce. We have described mechanisms that have been developed for this purpose; however, stress often leads to disease and death. This stress can be termed as “pathophysiological stress” and it is beyond the scope of this review. Moreover, bacterial and other infections consist a form of environmental stress that leads to neuronal inputs of immune responses, which have been discussed elsewhere (Rosas-Ballina and Tracey, 2009).

Despite the plethora of harsh conditions present in the life of an organism, living systems are remarkably adept at coping with internal and external stress and preserve their homeostatic balance. A big part of this ability relies on the nervous system as we described in this review. As neurons sense fluctuations in conditions, they use this information to orchestrate appropriate defense and adaptation responses at different levels (Figure 2). In addition, neurons can systemically act on other cells to regulate their response to stress. The importance of the role of the nervous system in stress responses is simply highlighted by the well-known ability of neurons to quickly respond to environmental changes. This ability integrates external inputs to the several different lines of defense that span from cell protection to behavioral strategies. The extent to which neurons have adapted to different stresses during their development is also demonstrated by the fact that developmentally immature neurons are more resistant to hypoxia (Romero et al., 2003; Shirai et al., 2006) than their mature counterparts, whereas mature neurons are more resistant to UV and IR than immature ones (Bickler and Buck, 1998). This resistance of neurons to different stresses at different developmental stages may reflect the exposure of the organisms to these stresses in the course of their life.

There is also an inherent element of stress in natural systems that is connected to aging. Regardless of how perfect an environment is and how well an organism is adapted to this environment, eventually the organism will functionally decline. Instead of considering aging as an event that comes late in the biological “equation” of living systems, we propose that aging is an extra layer of stress that applies throughout life and is related to the physical entropy (Figure 1). As energy cannot be transformed from one form to another without a qualitative loss (second law of thermodynamics), every single chemical reaction performed by a cell may contribute to aging. Similar ideas have been supported by others (Hayflick, 2007 and references therein).

Currently, aging is defined as a post-reproductive process precluding the selection for and transmission of the relevant genes to the next generation. However, a number of studies have now shown that many molecular pathways have an impact on organismal longevity and that these pathways are conserved among species. Our hypothesis that aging occurs before, during and after reproduction, could provide a basis for the evolution of a genetic program regulating aging. At the same time, the stochastic nature of aging due to physical entropy could explain the variations in lifespan and in expression of biomarkers of aging between genetically identical individuals, for example in cloned worm populations (Herndon et al., 2002). However, other explanations have been provided for such variations like epigenetic modifications occurring in early development and adulthood (Bell and Spector, 2011; Steves et al., 2012).

In addition to the active neuronal responses under certain stresses, neurons can experience several side effects under stressful conditions. The distinction between an active neuronal response and a stress-derived neuronal defect is often not clear. In the laboratory environment a potential beneficial advantage of a neuronal phenotype under a stressor can be missed due to the absence of the condition under which this phenotype promotes survival, and which is present in the natural habitat of the organism. However, strong deteriorative impact of stress on different structures can be recognized in many cases as we described.

The study of intrinsic developmental stress and its significance to embryonic and neuronal development is admittedly technically challenging. Very few studies have clearly shown the impact of such stress on normal development (Zhang et al., 2011a) and fewer of them have focused on neurons (Benard and Hobert, 2009). However, the information derived from these studies provide the basis for further investigations and provide a first glance as to the importance of the different aspects of intrinsic developmental stress on normal development.

In the field of human and murine biology, “stress” has mainly been associated with the psychological reaction of an individual to stressful external stimuli, and has been connected to the central nervous system, which generates sentiments like fear and anxiety. Although this consists a very important aspect of the biology of “stress,” it is extremely complex in its origin and manifestation. Therefore, we think that studying primary “stress” in a reductionist manner using models like *C. elegans* will provide us with more detailed information on how neurons survive and respond under “stress,” and how they are affected by it. Supportive of this approach are many examples where the psychological stress involves similar mechanism to other more basic aspects of stress (Yao et al., 2007).

## Conflict of Interest Statement

The authors declare that the research was conducted in the absence of any commercial or financial relationships that could be construed as a potential conflict of interest.
